# Pollen Dispersal in Fragmented Populations of the Dioecious Wind-Pollinated Tree, *Allocasuarina verticillata* (Drooping Sheoak, Drooping She-Oak; Allocasuarinaceae)

**DOI:** 10.1371/journal.pone.0119498

**Published:** 2015-03-05

**Authors:** Linda Broadhurst

**Affiliations:** Centre for Australian National Biodiversity Research, National Research Collections, CSIRO, Canberra, Australia; University of New South Wales, AUSTRALIA

## Abstract

Vegetation clearing, land modification and agricultural intensification have impacted on many ecological communities around the world. Understanding how species respond to fragmentation and the scales over which functionality is retained, can be critical for managing biodiversity in agricultural landscapes. *Allocasuarina verticillata* (drooping sheoak, drooping she-oak) is a dioecious, wind-pollinated and -dispersed species with key conservation values across southeastern Australia. But vegetation clearing associated with agricultural expansion has reduced the abundance and spatial distribution of this species in many regions. Spatial genetic structure, relatedness among trees, pollen dispersal and mating patterns were examined in fragmented *A. verticillata* populations selected to represent the types of remnants that now characterise this species. Short scale spatial genetic structure (5–25 m) and relatedness among trees were observed in most populations. Unexpectedly, the two male trees closest to each female did not have a reproductive advantage accounting for only 4–15% of the seed produced in larger populations. Biparental inbreeding was also generally low (<4%) with limited evidence of seed crop domination by some male trees. More male trees contributed to seed crops in linear remnants (mean 17) compared to those from patch remnants (mean 11.3) which may reflect differences in pollen dispersal within the two remnant types. On average, pollen travels ~100 m irrespective of remnant type but was also detected to have dispersed as far as 1 km in open landscapes. Low biparental inbreeding, limited reproductive assurance for near-neighbour and probably related males and variability in the distances over which females sample pollen pools suggest that some mechanism to prevent matings between relatives exists in this species.

## Introduction

Vegetation clearing, land modification and agricultural intensification have severely depleted and fragmented many ecological communities in temperate and tropical regions. Globally, these changes have resulted in 10–20% of drylands becoming degraded, forests disappearing in 25 countries, and inland water ecosystems now being in worse condition overall than any other broad ecosystem [1]. Consequently, this loss and degradation is now impacting on biodiversity, food and energy security, human health and well-being, and water availability [2]. Understanding how changes to vegetation structure and abundance influence the persistence of plant populations has been an important research focus for more than three decades, resulting a large body of empirical and theoretical knowledge. The long term persistence of remnant plant populations can be compromised by genetic erosion and increased inter-population genetic divergence through founder events, increased genetic drift, elevated inbreeding, reduced inter-population gene flow, and an increased likelihood of local extinctions [3]. Low genetic diversity may also limit the capacity of populations to adaptively respond to environmental change [4–6]. Understanding how plant populations respond to fragmentation at local and broad spatial scales is important to help land managers make strategic decisions regarding the conservation and management of populations, species and ecological communities. This is especially important in landscapes where tension exists between maintaining agricultural productivity and retaining biodiversity values.

One of the major changes imposed by fragmentation on plant populations is a shift to increased selfing with associated inbreeding consequences mostly seen as reduced seed production and/or less vigorous seedlings [7–12]. Trees are predicted to be more resilient to the effects of small population size and increased isolation following fragmentation due to their longevity, abundance, high reproductive capacity and ability to rapidly colonise new habitats [13,14]. But high outcrossing rates, large dispersal distances and long generation times can limit our ability to detect the signals of fragmentation in trees [15,16]. Fragmentation may also be less important for wind-pollinated and-dispersed trees since these do not rely on biotic vectors whose abundance and behaviour can be altered following changes to vegetation structure [17–19]. Although pollen and seed of wind-dispersed species are often presumed to regularly travel over long distances, counteracting negative inbreeding effects induced by fragmentation [20,21], some studies indicates that this is not necessarily the case [22,23]. Evidence does exist to suggest that a lack of obstacles in fragmented landscapes may be facilitating long distance dispersal [24]. However, bimodal pollen dispersal that includes highly localised (within tens of metres) and long distance (several kilometres) movement, is more likely to be characteristic of wind-reliant species [25], with short spatial genetic structure (40–100 m) indicating highly localised seed movement in these species [26–28]. Other reported effects associated with fragmentation for wind-pollinated species include reduced pollen availability and preferential near-neighbour matings [23,29–31]. These data suggest that despite perceptions of wind-reliant species being more robust to the effects of fragmentation, risks associated with inbreeding do exist. Indeed, there is now increasing evidence that reproductive output and progeny fitness in these species is being negatively impacted by fragmentation [11,29,32–38].

**Fig 1:**
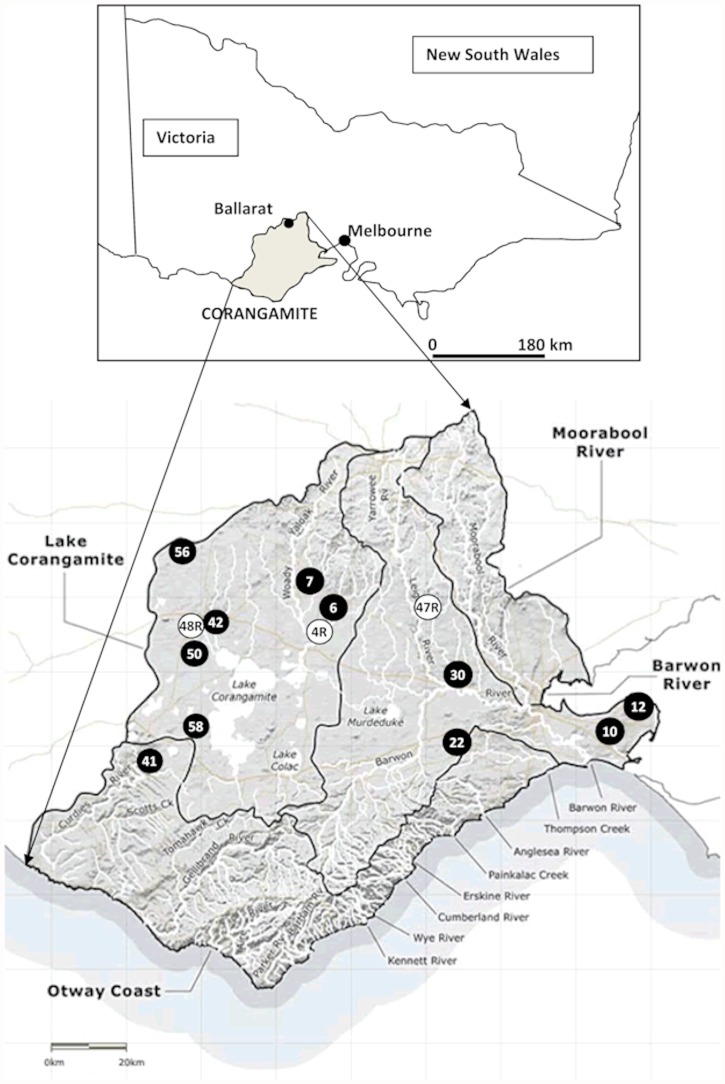
Location of *Allocasuarina verticillata* populations from the Corangamite Catchment (shaded in grey) in southeastern Australia. Numbers correspond to populations in Table 1; white circles indicate restored sites.

For dioecious species, however, inbreeding via selfing is impossible, eliminating this as a mechanism for fragmentation-induced negative effects. Dioecy does not, however, preclude matings between closely related trees (biparental inbreeding) and several studies indicate that this type of inbreeding can have negative consequences on progeny traits and seed set in wind-pollinated species [38–41], although this can be somewhat lineage dependent [42–44]. Demographic variables such as tree height, phenology, population density, individual variability in fecundity as well as wind direction also influence reproduction in these species [30,45–48]. These data indicate that the effects of fragmentation on wind-reliant species are likely to be complex. This study aims to understand the effects of fragmentation on localised pollen dispersal in the dioecious and wind-pollinated and-dispersed tree, *Allocasuarina verticillata*. This once broadly distributed species has been extensively fragmented by vegetation clearing during agricultural expansion across southeastern Australia. The research was conducted in the Corangamite Catchment in Victoria (Fig. 1) using *A. verticillata* populations selected to represent the various types of remnant vegetation left following land clearing. These remnants can typically be classified as small and isolated, linear road verges and relatively large intact patches. Three restored populations were also included as these should be maturing in to self-sustaining entities producing responses that resemble those observed in natural populations. The study populations are part of a larger research program to help prioritise conservation efforts and guide the selection and movement of seed for restoration projects by assessing genetic diversity and population genetic structure [49], examining mating patterns and pollen dispersal (reported here), and determining the consequences of fragmentation on germination and growth of seed crops (underway). This research component specifically sought to determine pollen dispersal patterns in the study populations and investigate potential drivers for observed responses, including the role of remnant type (i.e. small, linear and patch). Firstly, spatial genetic structure (SGS) and relatedness were determined for each population as this can be a major influence on the success of pollination events. Secondly, paternity assignment was used to evaluate the role of near-neighbour male trees in pollination success, assess how many males contribute to seed crops, whether any male dominance was occurring and determine the distances over which successful within-population pollination events were occurring. Finally, biparental inbreeding was used to assess whether this was likely to be a major influence on seed crop fitness and long term population persistence. Collectively, these data were used to understand the likely effects of fragmentation on seed crops and population persistence of fragmented *A. verticillata* populations.

**Table 1:** Population and sampling information and mating system and relatedness data for *Allocasuarina verticillata*.

				No. samples genotyped					No. related trees		
Remnant type	ID	Name	Pop size	Female	Male	Seed	*t*_m_-*t*_s_ (s.d.)	*r*_p_ % (s.d.)	M-M	M-F	% related
**Small**	41	Bostock Creek	7	2	5	17	0.004 (0.001)	0 (0.0)	1	0	14.3
	42	Lismore Cemetery	6	4	2	125	0.002 (0.013)	8.8 (3.1)	0	0	0
	50	Kinlock	6	3	3	66	0.006 (0.003)	31.4 (11.8)	0	0	0
	56	Ascott Downs	3	1	2	47	0.000 (0.000)	0.6 (0.0)	*	*	*
	58	Pomborneit North	13	6	6	89	0.031 (0.016)	13.9 (4.8)	0	2	16.7
**Linear**	6	Skipton	50	10	20	178	0.051 (0.013)	6.1 (1.3)	4	9	43.3
	12	Grassy	50	10	20	157	0.044 (0.018)	16.2 (2.5)	10	13	76.6
	22	Buckley	400	10	21	153	0.096 (0.026)	11.0 (3.0)	7	9	51.6
**Patch**	7	Cape Clear	500	9	23	47	0.116 (0.055)	7.4 (4.5)	9	5	45.2
	10	Blairwood	1000	10	19	87	0.034 (0.011)	3.7 (1.0)	3	4	24.1
	30	Wallace	6000	10	19	85	0.003 (0.001)	1.9 (0.8)	7	12	65.6
**Restored**	4R	Stevens	30	8	15	44	0.078 (0.025)	8.8 (6.5)	3	7	43.5
	47R	Nolan	50	8	19	47	0.001 (0.002)	21.8 (7.4)	8	15	85.1
	48R	Titanga	18	4	8	17	0.000 (0.000)	0 (0.0)	1	1	16.7

*t*_m_-*t*_s_, biparental inbreeding; *r*_p_ (%), correlated paternity; M-M, number of male-male relationships; M-F, number of male-female relationships; % related, percentage of sampled trees that are related; (s.d.), standard deviation; *, too few samples for analysis.

## Materials & Methods

### Species background, study sites and sampling

*Allocasuarina verticillata* (drooping sheoak, drooping she-oak) is a dioecious wind-pollinated and-dispersed small tree (5–9 m) widely distributed across southeastern Australia. It can tolerate a range of unfavourable environmental conditions [50] and is an important food source for Glossy Black-Cockatoos (*Calyptorhynchus lathami halmaturinus*). This subspecies is restricted to Kangaroo Island in South Australia and protected under the Australian *Environmental Protection and Biodiversity Conservation Act* 1999 [51,52] as well as South Australian state legislation. In addition, *C. lathami* is considered to be Vulnerable in Queensland, Victorian and New South Wales. Consequently, *A. verticillata* is a key restoration species providing multiple benefits in agricultural regions. This study was undertaken in the Corangamite catchment which covers ~13,000 square km in south-western Victoria (Fig. 1, [53]). Land clearing in the catchment has reduced natural vegetation by ~75%, leaving as little as 5% in some areas, making restoration a high priority [53]. Remnant and restored *A. verticillata* populations (Fig. 1, Table 1) were surveyed during spring 2006 and 14 populations representing the types of remnants present within the Catchment and more broadly across southeastern Australia were selected for study. Populations were categorized as small and isolated (<10 trees, 5 populations), linear road verges (3 populations) or large patches (3 populations). Of the small populations, Ascot, Bostock and Pomberneit contained tightly clumped trees whose close spatial proximity prevented individual GPS readings being obtained while Lismore trees were within 100 m of each other. Two small groups of trees (2 and 4 trees) ~1 km apart in a very open landscape with no other *A. verticillata* trees within the vicinity were sampled at Kinlock. All seed from Kinlock was later assigned with high confidence (90%) to the two males trees in these groups and all trees were subsequently combined as a single population. Trees at the linear Skipton population were distributed over ~400 m while six clumps over 2 km were sampled at Grassy. Buckley was a long thin triangle ~ 500 m on the longest edge and 100 m at the base. Patch populations also varied with Cape Clear and Wallace being rectangles (100 x 200 m and 400 x 150 m respectively) while Blairwood trees were patchily distributed over >2 ha with denser populations lining the nearby road verge. Trees at the restored Nolan, Titanga and Stevens populations were linearly distributed over 350, 400 and 500 m respectively. All sampling on private property (Stephens, Nolan and Titanga) was conducted with permission of the relevant landholders. Sampling at other sites was undertaken using field permits issued to Greening Australia staff in accordance with Victorian State Government regulations or were sampled by Department of Sustainability and Environment Victoria staff as the relevant land management authority. This study did not include sampling of any endangered or protected species.

Cones were collected from around the canopies of up to ten randomly chosen female trees in each population to extract seed. Cladodes were also collected from these females and their two closest males under the assumption these were most likely to dominate the pollen pools of their neighbouring females. All sampled trees were mapped to GPS locations. Cladodes were kept cool and lyophilised for storage prior to DNA extraction. Seed were extracted from dried cones, sub-sampled, germinated in petri dishes under laboratory light and temperature conditions and seedlings lyophilised for later DNA extraction. Germination was poor in some seed collections limiting the number of samples available for analysis. Trees at most populations appeared to be of the same cohort with little evidence of regeneration at most sites.

### DNA extraction and amplification

Genomic DNA was extracted from the cladodes of all sampled trees as well as up to 15 seedlings from each female [54] and amplified in 5μL reactions with six microsatellite primer pairs [49]: 1 X PCR buffer (Invitrogen), 5 μM of each dNTP (Sigma), 0.05 μM of each forward and reverse primer (forward primers were fluorescently labelled at the 5′-end with FAM from Thermo Scientific or NED, PET or VIC from Applied Biosystems), 5U/μL Platinum *Taq* (Invitrogen), 3 mM MgCl_2_ (Invitrogen)], 1 M Betaine (Sigma)] and 10–30 ηg template DNA with an Eppendorf Mastercycler. Amplicons were visualised on a 3130XL sequencer (Applied Biosystems) with a LIZ 600bp internal standard and scored with GeneMapper Version 4.0. Null allele frequencies and significant deviation from Hardy-Weinberg expectations were estimated for each site using CERVUS Version 3.0 [55].

### Spatial genetic structure and relatedness

Since mating patterns can be influenced by spatial structure within populations, spatial autocorrelation was undertaken using the genetic-distance-based, multivariate approach developed by in GenAlEx Version 6.41 [56,57]. All linear, patch and restored populations were assessed, however, most small populations were too clumped or had too few trees for this analysis with the exception of Kinloch. Pairwise genetic distances were estimated from the data while geographic distances were calculated from the GPS location of each tree. Since the choice of distance class can influence the detection of spatial genetic structure [58], variable distances classes (5, 10, 25, 50, 100, 250 and 500 m) were used as this approach is less prone to parameter errors and may be more reliable in estimating the spatial extent of genetic structure [59]. For the longer Grassy population additional distance classes of 1000, 1250, 1500, 1750 and 1950 m were used. Correlegrams of the estimated autocorrelation coefficient (*r*) were produced with the significance of *r* tested by 9999 random permutations to generate 95% confidence intervals about the null hypothesis of no spatial genetic structure (*r = * 0). Significant spatial structure was inferred at the distance where *r* fell outside the 95% confidence intervals which were estimated via bootstrapping (9999) [58].

As groups of highly related trees can lead to inbreeding, relationships among adult trees were assessed using ML-RELATE [60]. A conservative approach was adopted for this analysis whereby null alleles were assumed with relatedness coefficients (*r*) estimated by maximum likelihood over 10,000 randomisations. Relationship categories were then estimated for each population with trees being assigned as half-sibs, full-sibs, parent-offspring and unrelated (p<0.05). Confidence intervals of pairwise relatedness and relationships between trees were then estimated over 10,000 randomisations. Since there was no way to biologically validate relationships among adults trees (e.g. through known parent-offspring or sibling relationships), the data were conservatively categorised as either related (i.e. half-sibs, full-sibs, parent-offspring) or unrelated. These relationships were later used to determine the number of unambiguously assigned seed that had resulted from matings between related or unrelated trees.

### Pollen movement and mating patterns

**Paternity assignment**. Pollen donors were assigned using the maximum-likelihood approach in CERVUS Version 3.0.3 [55]. CERVUS uses multi-locus genotypes to calculate a likelihood ratio for each candidate parent-offspring pair, then estimates the difference in LOD scores between the most likely and the next most likely parent. Confidence intervals were estimated using simulations of parental and offspring genotypes with the observed allele frequencies using the parameters of 100,000 cycles, 0.01 mistyping error, and for strict and relaxed confidence intervals specified as 90% and 85% respectively. The number of males genotyped at each population were considered to be the potential fathers with 90% assumed to be sampled in the small, revegetation and two of the linear populations (Skipton and Grassy) whereas 70% were assumed to have been sampled in the larger (>50 plants) populations. Progeny were categorised as matings between a female tree and one of the two nearest males, matings with other sampled males within the population and matings with males that could not be assigned (unsampled males).

**Pollen dispersal**. Ideally, pollen dispersal curves would provide additional information regarding the pollen movement for these populations but TwoGener assumes that plants are regularly spaced and phenologically synchronized [61]. Since plant density was often clumped and phenological synchronicity could not be validated both of these assumptions were assumed to be violated, potentially leading to an inflated Φ_ft_. Accordingly, minimum pollen dispersal distances were calculated by averaging the distance from each female tree to the assigned pollen donors. Bubble plots of the mean pollen dispersal distance for female trees in each population were generated to assess whether location within a population was related to the distance over which pollen was being sampled. Pollen distances were also averaged for each remnant type.

**Biparental inbreeding and correlated paternity**. Maximum likelihood estimates of single- (*t*_s_) and multi-locus (*t*_m_) outcrossing rates were calculated following the mixed-mating model with female genotypes included [62]. Since for dioecious species outcrossing must equal 1, these values were only estimated to enable calculation of biparental inbreeding (matings among relatives, *t*_m_-*t*_s_). Correlated paternity (*r*_p_), which estimates the proportion of a female’s progeny that were sired by the same male, was calculated using the sibling-pair model in which *r*_p_ is the probability that two individuals drawn at random from the same progeny array are full-sibs [63]. All parameters were calculated using the Newton-Raphson method for six loci in MLTR version 3.4. Standard errors were generated from 500 bootstraps with resampling among female trees and unpooled pollen and ovule frequencies [64,65].

## Results

### Null alleles and Hardy Weinberg deviations

There was no evidence of null alleles but some significant Hardy-Weinberg (HW) deviations were associated with some loci and populations. These were, however, inconsistent across loci and populations and all loci were retained for analysis.

### Spatial genetic structure and relatedness

**Fig 2:**
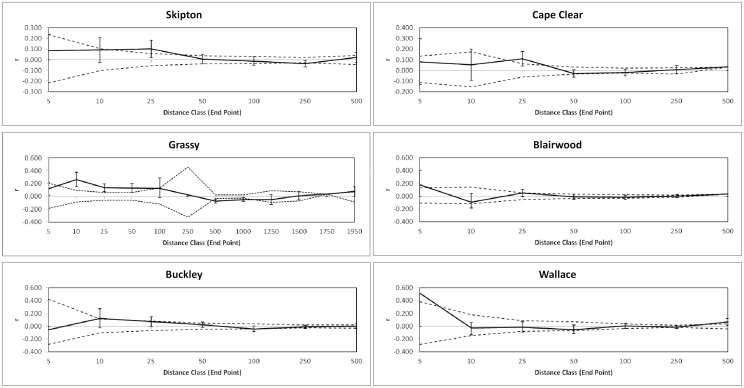
Spatial autocorrelation coefficient values (*r*) for *Allocasuarina verticillata* populations where significant structure was observed. Dashed lines indicate upper and lower 95% CI around *r* based on 9999 simulations; 95% confidence bars around *r* from 9999 bootstraps. Linear populations left panels, patch populations right panels.

No significant structure was found at the small Kinlock or restored populations. The remaining correlograms indicated that significant positive genetic structure existed in the linear and patch populations but that the scale over which this occurred varied (Fig. 2). For example, significant structure was evident at 25 m for Skipton (*r*_ij_ = 0.102, P = 0.042) and Cape Clear (*r*_ij_ = 0.109, P = 0.001) whereas at Buckley this was significant at both 10 m and 25 m (*r*_ij_, = 0.123, P = 0.017 and *r*_ij_ = 0.073, P = 0.033 respectively). Blairwood also exhibited significant structure at two distances (5 m, *r*_ij_ = 0.175, P = 0.007 and 25 m, *r*_ij_ = 0.049 P = 0.041) while four distances were significant in the very long linear Grassy population [10, 25, 50 m and 100 m (*r*_ij_ = 0.262, *r*_ij_ = 0.135, r = 0.131 and *r*_ij_ = 0.127 respectively; first three values all P<0.001, last value P = 0.030). In contrast, spatial structure existed at very small scales in the Wallace patch (5m, *r*_ij_ = 0.511, P = 0.006).

Relatedness among adults was observed in most of the natural populations varying from a total of seven relationships at Blairwood to 23 along Grassy (Table 1). The number of relationships among restored trees also varied with almost all of the Nolan trees being related in contrast to only two of the Titanga trees. Generally more male-male relationships were detected but this probably reflects the 2:1 male-female sampling strategy providing greater probability to detect male-male relationships. The number of male-female relationships also varied among populations ranging from four relationships at Blairwood to 13 along Grassy.

### Pollen movement and mating patterns

**Fig 3:**
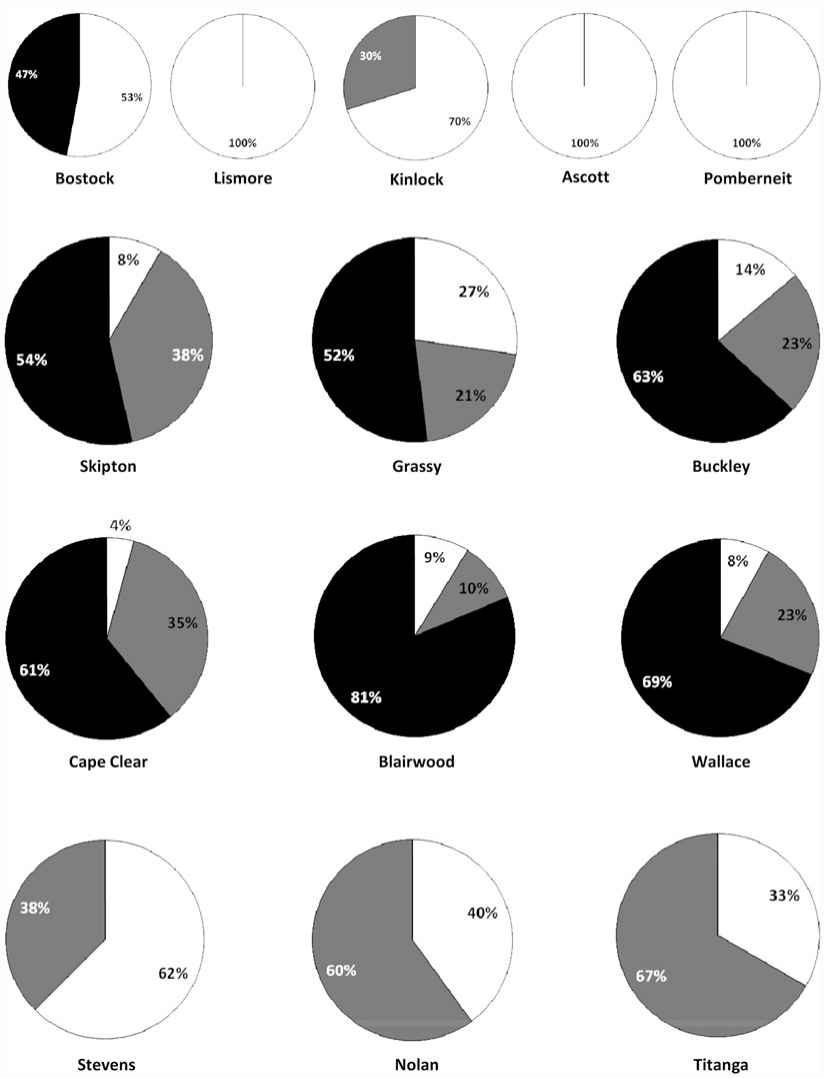
Proportion of *Allocasuarina verticillata* seed crops that could be ascribed to near-neighbour males (white), other sampled males (grey) and unsampled males (black). Pie charts ordered in rows top to bottom as small, linear, patch and restored populations.

**Paternity assignment**. Seed paternity could be assigned with high confidence (90%) in small and restored *A. verticillata* populations, indicating the utility of these markers for this study. Fig. 3 indicates the proportion of each seed crop that could be assigned to the two nearest male trees, other sampled males within the population and to unsampled males. All of the seed from the small Lismore, Ascott and Pomberneit populations were sired by the two nearest males. In contrast, 47% of Bostock seed was pollinated by males external to this population while 30% of Kinlock seed was assigned to other sampled males. Nearest males contributed to only 8–27% of the seed crops in linear populations with a further 21–38% being assigned to other sampled males. But the majority of seed in these populations was fertilized by unsampled males (52–63%). The proportion of progeny sired by near-neighbour male trees in patches was consistently low (4–9%) while seed assigned to other sampled males ranged from 10–35% with the remaining 61–81% being from unsampled males. Seed from restored populations were only sired by near-neighbour (33–62%) or other sampled male trees (38–67%).

**Fig 4:**
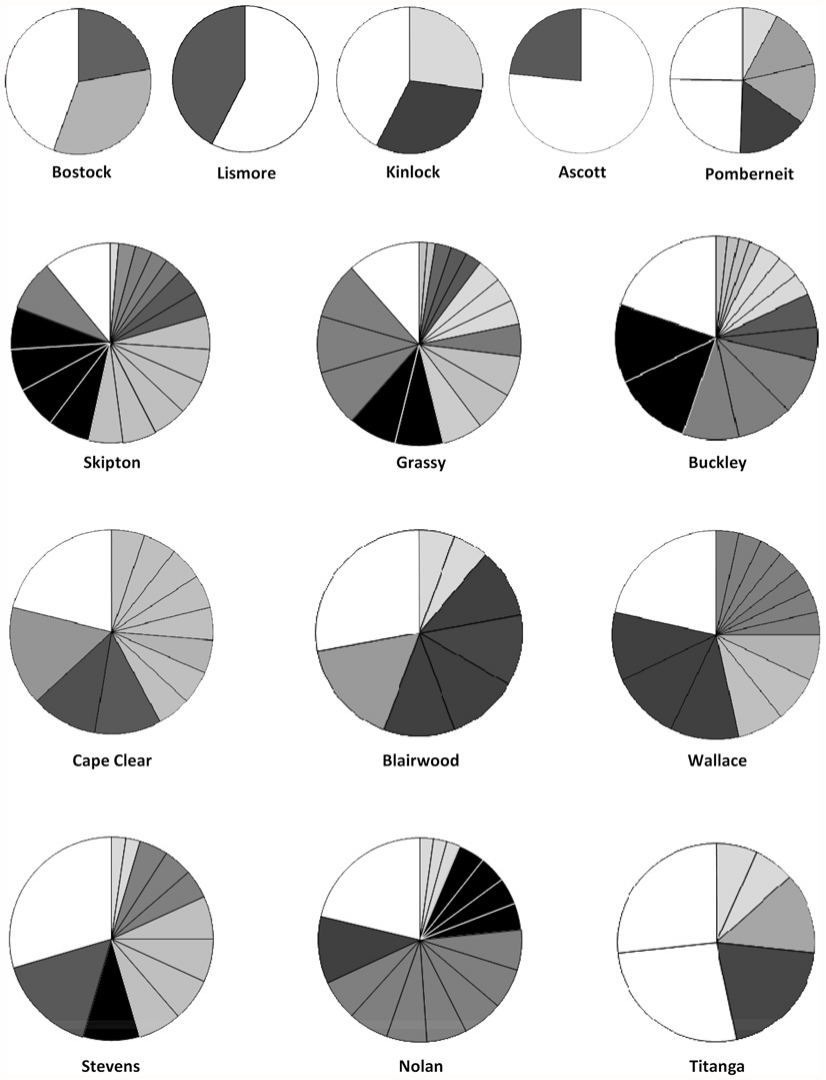
Relative proportion of male tree contribution to the assigned portion of seed crop from each *Allocasuarina verticillata* population. Pie slices with the same colour indicate the same percentage contribution from several male trees. Pie charts ordered in rows top to bottom as small, linear, patch and restored populations.

Male contribution to the assigned portion of seed crops at each population was highly variable (Fig. 4). For example, three males fertilized 44% of the assigned seed crop in the linear Buckley whereas contributions were more evenly distributed among males at the other linear populations (Skipton and Grassy). In patch populations, seed crop domination occurred at Cape Clear with four males siring 58% of the seed, two Blairwood males fertilized 44% of the seed and four Wallace males accounted for 53%. Almost 50% of sampled Cape Clear and Blairwood males made no contribution to the seed crops at all. Some male dominance was also evident in restored populations with two males siring 45% of Stevens and 34% of Nolan seed crops with three Titanga males accounting for 73%. Small populations also showed male bias in their seed crops, most especially at Lismore and Ascott where a single male contributed to 58% and 77% of the seed crops respectively. On average, 17 males contributed to linear remnant seed crops as opposed to 11.3 males in both patch and restored populations.

**Fig 5:**
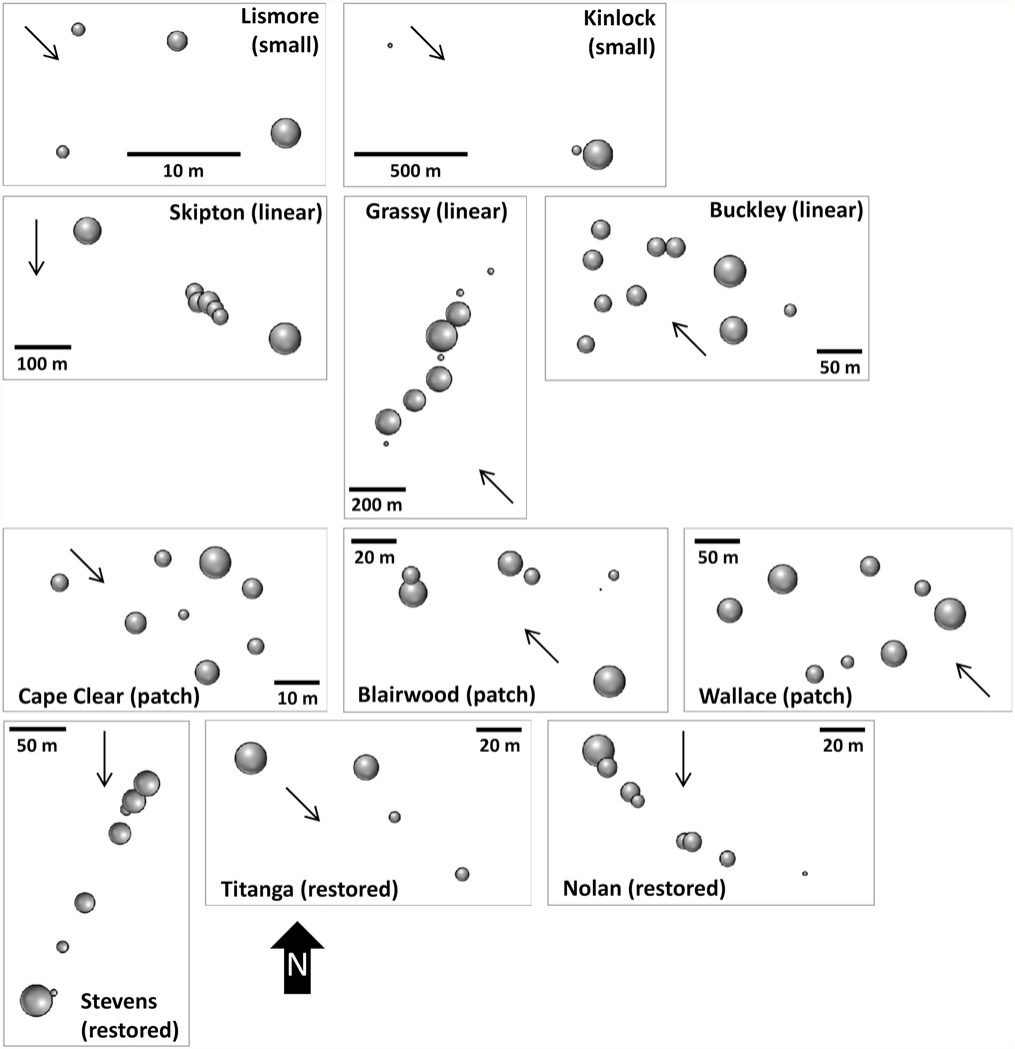
Proportional bubble plots for each *Allocasuarina verticllata* population to indicate the mean distance over which female trees are sampling pollen. Bubbles indicate that spatial location of each mother tree. The larger a bubble, the longer the mean distance over which pollen has been sampled. Arrows within plots indicate the predominant wind direction during flowering from the nearest meteorological station.

**Pollen dispersal**. Mean pollen distances for the four remnant types were 200 m for small populations (109 m when the widely distributed Kinlock population was removed), 105 m for linear remnants, 107 m for patches and 70 m for restored populations. Fig. 5 illustrates the mean distance over which female trees sampled pollen with longer mean distances represented by larger bubbles. Within populations these distances were highly variable and did not appear to correspond with where a female was located. For example, at Lismore and Kinlock some peripheral female trees sample pollen more broadly (i.e. have larger bubbles) which appeared to reflect predominant wind direction. But overall there were no clear patterns associated with the location of a female, the distance over which pollen was sampled and predominant wind direction during flowering.

**Biparental inbreeding and correlated paternity**. As expected of a dioecious species outcrossing rates were 1.00. Levels of biparental inbreeding were relatively low at most populations (range 0.000–0.051, Table 1) but were higher at the restored Stevens (8%), linear Buckley (~10%) and Cape Clear patch (~12%) populations. Correlated paternity was <11% at ten of the 13 populations but was higher at Pomberneit (14%) and Grassy (16%) and much higher at Nolan (22%). Correlated paternity was highest at Kinlock where 31% of the seed crops from each mother was sired by a single pollen donor (male tree). The percentage of seed resulting from matings between these related trees ranged from none to low in eight populations, 13–16% at Pomberneit, Skipton and Buckley and to as high as 23–25% at Grassy and Wallace.

## Discussion

Short spatial genetic structure (5–25 m) characterised many *Allocasuarina verticillata* populations, irrespective of remnant size or shape, with most of these also comprised of related trees. Contrary to expectations, near-neighbour males did not have a reproductive advantage in terms of pollination success with female trees generally sampling pollen from males within populations at least~70–100 m. Biparental inbreeding was low across all populations while correlated paternity was variable (range 0–31%) with some seed crops being dominated by relatively few males. There was also strong evidence that pollen was travelling more than 1 km in landscapes with few obstacles.

The finding that the two *A. verticillata* males trees closest to each female did not fertilise the majority of her seed contrasts strongly with other wind-pollinated species where >40% of observed matings were by near-neighbours [30,31]. Even in small *A. verticillata* populations where low mate availability should increase the likelihood of near-neighbour matings, male proximity did not necessarily guarantee success. For example, 47% of seed at Bostock was fertilized by immigrant pollen while 30% of Kinlock seed was sired by distant rather than nearby males. In larger populations, near-neighbour male trees accounted for only 4–15% of the seed produced. Why *A. verticillata* males should have so little influence on the seed crops of their nearest female tree is unclear, but one possible explanation is that relatedness among adult trees is influencing fertilization success. The fine scale non-random spatial genetic structure (5–25 m) and levels of relatedness observed among these *A. verticillata* trees (mean relatedness 57%, and 45% in linear and patch populations respectively) have been observed in other dioecious and wind-pollinated plants [20,26–28,36].

Plants have evolved several mechanisms to limit inbreeding following selfing and/or matings with close relatives (biparental inbreeding). For dioecious species, inbreeding via selfing is precluded by the separation of male and female gametes to different plants, but biparental inbreeding is possible unless some mechanism to eliminate or prevent these matings exists. Generally low biparental inbreeding (<5%), limited pollination success by near-neighbour males and variability in the distances over which female *A. verticillata* trees sample pollen suggest that females may be excluding pollen from related males perhaps through some type of incompatibility system. Similarly low biparental inbreeding has been observed in other wind-pollinated species such as *Nothofagus nervosa* [4%, [66]] and *Vochysia ferruginea* [4%, [67]] but whether this signals that these species, as well as *A. verticillata*, have some mechanism to avoid biparental inbreeding is unclear. However, reproductive strategies such as post-pollination selection to minimise inbreeding have been found in both wind-pollinated and dioecious species [39,68]. If *A. verticillata* does employ such a strategy, low biparental inbreeding values such as those observed here are to be expected as these matings are less likely to result in viable seed that can be sampled and analysed. While higher biparental inbreeding was present at the restored Stevens (8%), linear Buckley (10%) and patch Cape Clear (12%) populations it is unclear what may be influencing these results. These values were marginally lower than that observed in highly fragmented *Pistacia lentiscus* populations [14%, [69]] and may reflect that these *A. verticillata* populations are more isolated. It is also possible, however, that these data reflect the idiosyncratic nature of wind-pollination including year to year variation [70].

Pollination success in wind-pollinated species is influenced by many factors including wind direction, tree height, phenology, spatial structure and population density [30,45–47]. Measuring these parameters was beyond the scope of this study but all sampled *A. verticillata* tree were reproductively mature and similarly sized suggesting that tree height may not be a major influence of male reproductive success. Wind direction may be important but no site specific data exist for any of these populations. Phenology may also play a role but cones are the product of the flowering from the previous season, making it impossible to determine how much of a role phenology played in the results reported here. While pollination success can be somewhat idiosyncratic, of some interest here was the finding that *A. verticillata* pollen travels very similar distances (105–109 m) irrespective of remnant type. This suggests that if family structure and relatedness are driving pollination success, then this apparently breaks down at ~100 m. It is impossible to determine from these data whether the *A. verticillata* male trees responsible for the unassigned portion of each seed crop reside within or external to the linear and patch populations sampled here. But data from other wind-pollinated species indicates that more than 50% of seed crops [30,31,71] can result from immigrant pollen and up to 93% in highly fragmented *Pistacia lentiscus* populations [69]. Pollen assignment at the Kinloch population clearly show that *A. verticillata* pollen can travel at least 1 km in an open landscape where few obstacles exist which is consistent with distances observed in isolated and continuous populations of other wind-pollinated species [28,72]. There is some suggestion that increasingly open areas associated with vegetation removal may be facilitating long distance dispersal for wind-reliant species [24]. But it is unclear whether this is at levels high enough to counteract negative reproductive and progeny fitness effects observed in these species [11,29,32–34,37,38].

The contribution of individual male trees to seed crops in each population was highly variable as has been observed elsewhere [24,69]. But for *A. verticillata*, remnant type does appear to have some influence on the average number of males contributing to seed crops with more male trees contributing in linear populations (mean 17 males) than in patches (mean 11.3 males, Fig. 4). This result probably reflects differences in how pollen is dispersed among trees in the two remnants types. Trees in patches occur at lower densities and are more randomly distributed than those in long narrow linear remnants trees. Consequently, pollen clouds in patches may be less dense than in linear populations providing females in these latter populations with the opportunity to sample a more diverse pollen cloud [70]. Seed crop dominance by some males, 8–19 pollen donors in larger populations and moderate-high levels of correlated paternity at Buckley (11%), Pomberneit (14%), Grassy (16%), Kinlock (31%) and Nolan (22%) are in line with observations from other fragmented wind-reliant species [28,66,69]. It must be remember, however, that since the majority of seed in linear and patch populations (52–81%) were sired by unsampled males, the number of pollen donors in these seed crops is potentially higher. This suggests that seed crop domination by relatively few males and possible erosion of genetic diversity observed in other studies [73,74], is less of an issue in larger *A. verticillata* populations.

Restoring *A. verticillata* across southeastern Australia is an important component of conservation management not only for this species, but also for those that rely on it for food and shelter. While the success of restored populations is dependent on numerous factors, using seed that is highly related may limit the reproductive success of *A. verticillata* populations in the future. This may be especially important for the Nolan population where 85% of the trees were related and correlated paternity was one of the highest observed (22%). Seed for this site was sourced from several trees within 100 m highlighting one of the most detrimental issues to long term restoration success—the building of more, small populations that lack the genetic diversity required to become self sustaining entities [74]. Inbreeding and paternity responses in Titanga were similar to those in other small populations whereas Stevens exhibited one of the highest biparental inbreeding values (8%) with moderate correlated paternity (9%). A previous study of genetic diversity and population genetic structure indicted that seed from these restored populations could possibly be suitable seed sources in the future [49]. These data do not contradict these findings but do indicate that using highly related seed can be problematic for restoring this species. As recommended in the previous paper, seed from these restored populations should be mixed with other seed sources to increase overall genetic diversity and reduce the possibility of future matings between close relatives.

## Conclusions

Understanding how fragmented plant populations function both locally and across broad spatial scales is critical for improving conservation and management strategies. This may be especially so given that effective population sizes of ~1000 are predicted to maximise long term population persistence [75]. Despite expectations that fragmentation should have little effect on the persistence of wind-reliant species, this study adds to the growing body of evidence that suggests otherwise. Fine scale genetic structure and relatedness in fragmented *A. verticillata* populations appear to limit near-neighbour matings, possibly through some reproductive strategy to minimise inbreeding that requires further examination. Elevated inbreeding in this species is likely to impact on seed production and may have important consequences for animals such as Glossy Black-Cockatoos that rely on this species for food. Although pollen can travel at least 1 km across open landscapes this should not be considered as the minimum distance at which to connect *A. verticillata* populations since distance is negatively correlated with pollen immigration rates [28]. Indeed, the finding that pollen moves ~100 m on average within populations suggests that *A. verticillata* trees interact over much shorter distances. It also appears that linear remnants are not disadvantaged when compared to patches allowing land managers to construct new populations along fence lines and road verges in agricultural regions to reduce concerns associated with maintaining both productivity and biodiversity values in these landscapes. Overall, the long term future of this species in fragmented landscapes will likely depend on the amount, populations size, level of isolation and spatial structure of the remaining remnants. Future assessments of seed germination and seedling growth will provide further information to help evaluate the long term prospect for *A. verticillata*.
